# Toughening Mechanism of Unidirectional Stretchable Composite

**DOI:** 10.3389/frobt.2021.673307

**Published:** 2021-04-30

**Authors:** Xiaochun Jiang, Zhengjin Wang, Danqi Sun, Tongqing Lu, Tiejun Wang

**Affiliations:** State Key Lab for Strength and Vibration of Mechanical Structures, Soft Machines Lab, Department of Engineering Mechanics, Xi’an Jiaotong University, Xi’an, China

**Keywords:** stretchable composite, large deformation, interfacial debonding, stress concentration, toughness

## Abstract

Composite materials have been long developed to improve the mechanical properties such as strength and toughness. Most composites are non-stretchable which hinders the applications in soft robotics. Recent papers have reported a new design of unidirectional soft composite with superior stretchability and toughness. This paper presents an analytical model to study the toughening mechanism of such composite. We use the Gent model to characterize the large deformation of the hard phase and soft phase of the composite. We analyze how the stress transfer between phases deconcentrates the stress at the crack tip and enhances the toughness. We identify two types of failure modes: rupture of hard phase and interfacial debonding. We calculate the average toughness of the composite with different physical and geometric parameters. The experimental results in literature agree with our theoretical predictions very well.

## Introduction

Recently, the emergence of novel soft materials such as elastomers and gels, enable enormous applications including soft robots (Wallin et al., 2018; Whitesides, 2018), ionotronics (Lin et al., 2016; Wirthl et al., 2017; Yang and Suo, 2018; Yuk et al., 2019), stretchable electronics (Minev et al., 2015; Park et al., 2018), and wound dressings (Blacklow et al., 2019). These applications impose a big challenge for soft materials to improve their mechanical properties. Many natural materials such as shell, bone, wood, and muscle exhibit excellent mechanical properties in strength, toughness, and fatigue (Currey, 1977; Kamat et al., 2000; Fratzl and Weinkamer, 2007; Ji and Gao, 2010). These materials are all composite materials, made up of phases with different materials. The mechanical properties of the composite increase by orders of magnitude compared to each single phase (Kamat et al., 2000; Ji and Gao, 2010). Developing bioinspired stretchable soft composites is a promising solution to improve mechanical properties of soft materials.

Soft composites such as fiber-reinforced elastomers (Goettler and Shen, 1983) have been studied for many decades. Several recent papers reported fabric reinforced rubber or hydrogel composite with high strength and high toughness for potential use of soft robotics (Huang et al., 2017; Wang et al., 2018; Zhang et al., 2020). For all these soft composites, the fibers are stiff and non-stretchable, which sacrifices the stretchability of the composite. Until recently, Wang et al. and Xiang et al. discover that the stretchable hard phase can also reinforce the soft phase if the modulus ratio and interface bonding meet certain requirements (Wang et al., 2019; Xiang et al., 2020). They design a stretchable composite by periodically arranging hard phase and soft phase with strong adhesion in between. When the composite is stretched, the soft phase near the crack tip greatly shears, the hard phase is greatly stretched and stores most of the elastic energy. When the hard phase ruptures, the stored elastic energy is released. In this design, the composite can be stretched twice of the original length and the toughness of the composite can reach 10^3^–10^4^ J/m^2^. The composite also has a high fatigue threshold, low hysteresis, and low crack sensitivity. However, these works only report the experimental results and a reliable theoretical model to quantitatively analyze the stretchable composite is still lacking.

The existing theoretical models for unidirectional composites mainly focus on non-stretchable stiff fibers and adopt the theory of linear elasticity (Hedgepeth, 1961; Hedgepeth and Dyke, 1967; Hikami and Chou, 1990; Nedele and Wisnom, 1994; Beyerlein and Phoenix, 1996; Phoenix and Beyerlein, 2000; Swolfs et al., 2015; Hui et al., 2019). For example, Hedgepeth used the shear-lag model to analyze the load transfer process of the composite (Hedgepeth, 1961) and obtained the stress concentration factor of the first intact fiber near the crack tip. Hikami et al. calculated the stress concentration of multiple intact fibers near the crack tip (Hikami and Chou, 1990). Hui et al. treated the composite as an orthotropic plate and obtained an approximate solution of stress concentration at different crack lengths (Hui et al., 2019). To the authors’ best knowledge, no work has analyzed the deformation and stress concentration of highly stretchable soft composite.

In this work, we establish a theoretical model to analyze the stretchable composite with periodically arranged hard phase and soft phase. We use the Gent model to characterize the large deformation of hard phase and soft phase. We calculate the stress concentration of the composite and identify two types of failure modes: rupture of hard phase and interfacial debonding. We calculate the average toughness of the composite and compare the theoretical predictions with the experimental results in literature.

## Governing Equations

**FIGURE 1:**
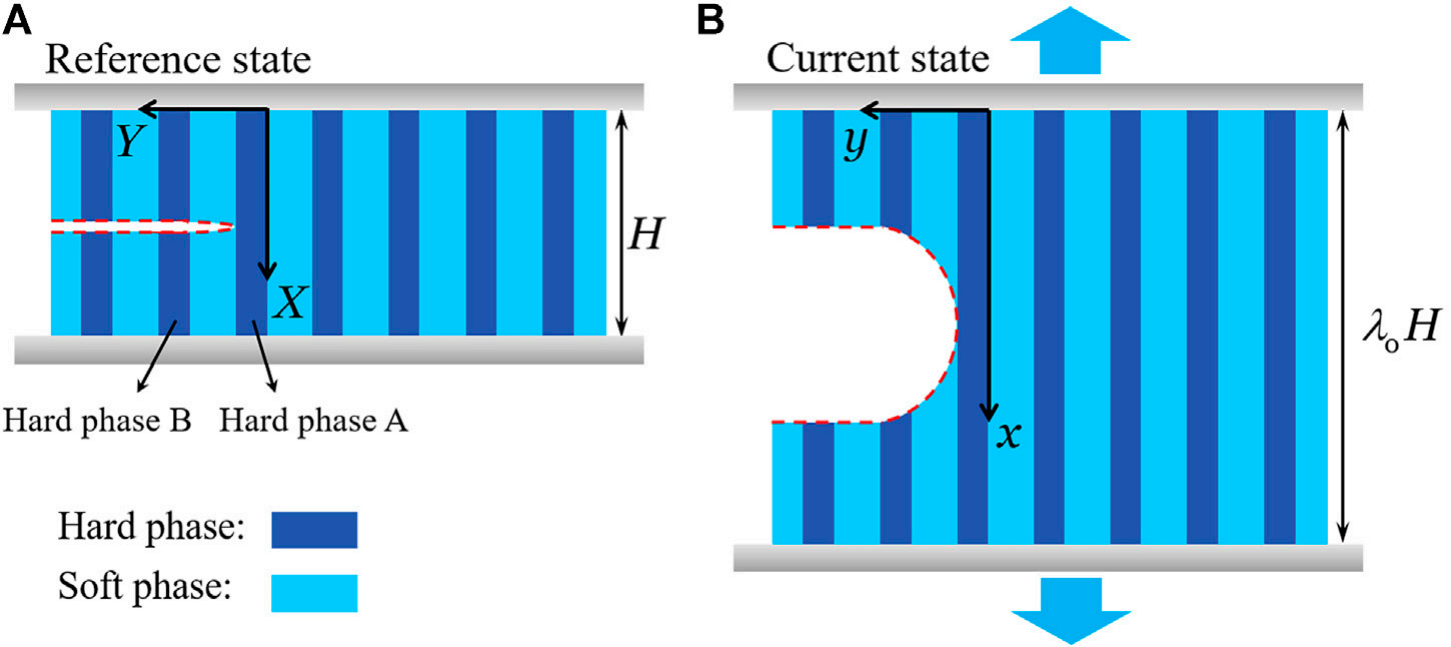
A stretchable composite consists of periodically arranged hard phase and soft phase, with the top and bottom ends clamped. **(A)** In the reference state, no force applies on the composite. A horizontal crack pre-exists in the middle. The coordinate is (*X,Y*). **(B)** In the current state, the composite is subject to a uniaxial displacement and the crack opens. The coordinate is (*x,y*).

Consider a stretchable composite consisting of periodically arranged hard phase and soft phase (Figure 1). The top and bottom ends of the composite are clamped. A horizontal crack pre-exists in the middle. The crack tip is at the interface of the hard phase and soft phase. In the undeformed state, the width of hard phase and soft phase are $w_{h}$ and $w_{s}$, and the thickness of hard phase and soft phase are $t_{h}$ and $t_{s}$. Subject to a vertical tension, the height of the composite changes from H to $\lambda_{0}H$. Consider the composite as a thin sheet and we simplify the problem as a plane-stress problem. The coordinate system is *X, Y* in the reference state (Figure 1A) and *x, y* in the current state (Figure 1B). The widths of the two phases, $w_{h}$, $w_{s}$ are much smaller than the height of the composite, *H*. Therefore, we consider the deformation field as a single function of *X* coordinate. We focus to analyze the deformation field of the three layers close to the crack, hard phase A, hard phase B, and the soft phase in between (Figure 1A). Define the displacement field of hard phase A right ahead of the crack tip as $u^{A}\left(X\right)$, the stretch as $\lambda^{A}\left(X\right)$, the nominal stress as $s^{A}\left(X\right)$.Define the stretch of hard phase B as $\lambda^{B}\left(X\right)$ and the nominal stress as $s^{B}\left(X\right)$. We assume the soft phase is only subject to simple shear and also neglect the *Y*-dependence of strain and stress. Define the shear strain and shear stress in the soft phase as γ(X) and τ(X).

When the composite deforms, the geometric relations are

$$\frac{du^{A}\left(X\right)}{dX}=\lambda^{A}\left(X\right)-1,$$ (1)

$$\frac{d\gamma \left(X\right)}{dX}=\frac{\lambda^{A}\left(X\right)-\lambda^{B}\left(X\right)}{w_{s}}.$$ (2)

Hard phase A is subject to a uniaxial tension and the shear stress from the soft phase. Hard phase B is subject to shear stress from the soft phase only. We apply the shear-lag model (Kaelble, 1960; Kaelble, 1992) to obtain the force balance of the two hard phases as

$$\begin{array}{l} \frac{ds^{A}\left(X\right)}{dX}=\frac{\tau \left(X\right)t_{s}}{w_{h}t_{h}},\\ \frac{ds^{B}\left(X\right)}{dX}=-\frac{\tau \left(X\right)t_{s}}{w_{h}t_{h}}. \end{array}$$ (3)

We use the Gent model to represent the stress-strain relations of hard phase and soft phase (Gent, 1996). In the hard phase, the tensile stress relates to the tensile stretch as

$$s=\frac{\mu_{h}\left(\lambda -\frac{1}{\lambda^{2}}\right)}{\left(1-\frac{\lambda^{2}+\frac{2}{\lambda }-3}{J_{\lim h}}\right)},$$ (4)

and in the soft phase the shear stress relates to the shear strain as

$$\tau =\frac{\mu_{s}\gamma }{\left(1-\frac{\gamma^{2}}{J_{\lim s}}\right)}.$$ (5)

The displacements of both hard phase and soft phase at the top and bottom ends are fixed, so we have

$$\begin{array}{l} {u^{A}\left(X\right)|}_{X=0}=0,\\ {\gamma \left(X\right)|}_{X=0}=0. \end{array}$$ (6)

At the middle of the hard phase A the displacement is equal to the half of the applied total displacement and the middle of the hard phase B is stress free. We have

$$\begin{array}{l} {u^{A}\left(X\right)|}_{X=\frac{H}{2}}=\frac{\left(\lambda_{0}-1\right)H}{2},\\ {s^{B}\left(X\right)|}_{X=\frac{H}{2}}=0, \end{array}$$ (7)

We solve the fields of displacement, stress, strain of the three layers combining Eqs 1–7 using MATLAB.

## Stress and Strain Analysis

In plotting the results, we use the representative geometric parameters as follows (Wang et al., 2019). The height of the composite is H=20 mm. The widths of hard phase and soft phase are $w_{h}=1\;\mathrm{mm}$, $w_{s}=2.125\;\mathrm{mm}$. The thicknesses of hard phase and soft phase are $t_{h}=0.5\;\mathrm{mm}$, $t_{s}=0.8\;\mathrm{mm}$. We fabricate PDMS samples (Sylgard 184 from Dow Corning) with five different curing ratios (weight ratio of base and curing agent m:n=10:1, 12:1, 15:1, 20:1, 30:1) and carry out uniaxial tensile tests to determine the material parameters (Table 1). The rupture stretches $\lambda_{c}$ for different samples are recorded and the parameters μ, $J_{\lim}$ in the Gent model are obtained by curve fitting (Supplementary Figure S1).

**TABLE 1:** Parameters of PDMS with different curing ratios.

Curing ratio m:n	μ(MPa)	$J_{\lim}$	$\lambda_{c}$
10:1	0.65	3.5	2.12
12:1	0.40	22.7	4.08
15:1	0.17	21.8	3.82
20:1	0.14	54.5	5.68
30:1	0.034	62.2	4.21

**FIGURE 2:**
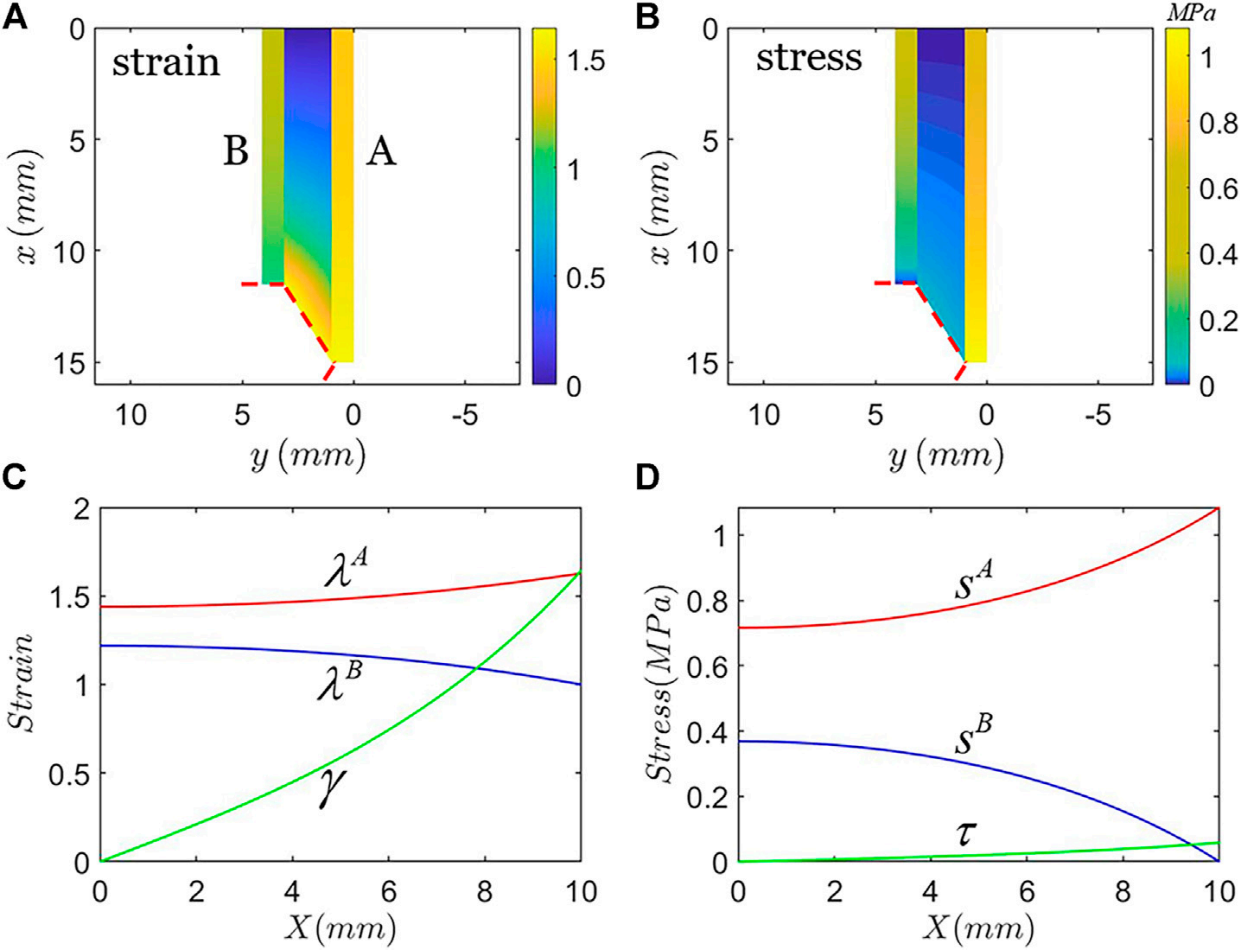
Strain field and stress field in the three layers close to the crack when the applied remote stretch is $\lambda_{0}=1.5$. Due to symmetry, only a half of the three layers is plotted. **(A)** Strain contour and **(B)** stress contour of the three layers plotted in the coordinate of the current state. **(C)** Strain distribution (tensile strain in the two hard phases and shear strain in the soft phase) and **(D)** stress distribution of the three layers plotted in the coordinate of the reference state.

Figure 2 plots the strain and stress distribution of the three layers close to the crack tip. The material of hard phase A is taken to be PDMS of weight ratio m:n=10:1 and the material of hard phase B is taken to be PDMS of weight ratio m:n=30:1. The total applied stretch to the composite is modest, $\lambda_{0}=1.5$. Figure 2A shows the strain of the composite in the current state and Figure 2C shows the strain in the reference state. In hard phase B, the strain gradually decreases along the x-axis. In both hard phase A and the soft phase, the strain gradually increases and reaches the maximum at the crack tip X=H/2. Figure 2B and Figure 2D show the similar trend of stress distribution in the current and reference state. At the crack surface, the stress reaches zero and the stretch is close to 1. We have checked that the difference in stress distribution is small if the calculations include more layers than three.

**FIGURE 3:**
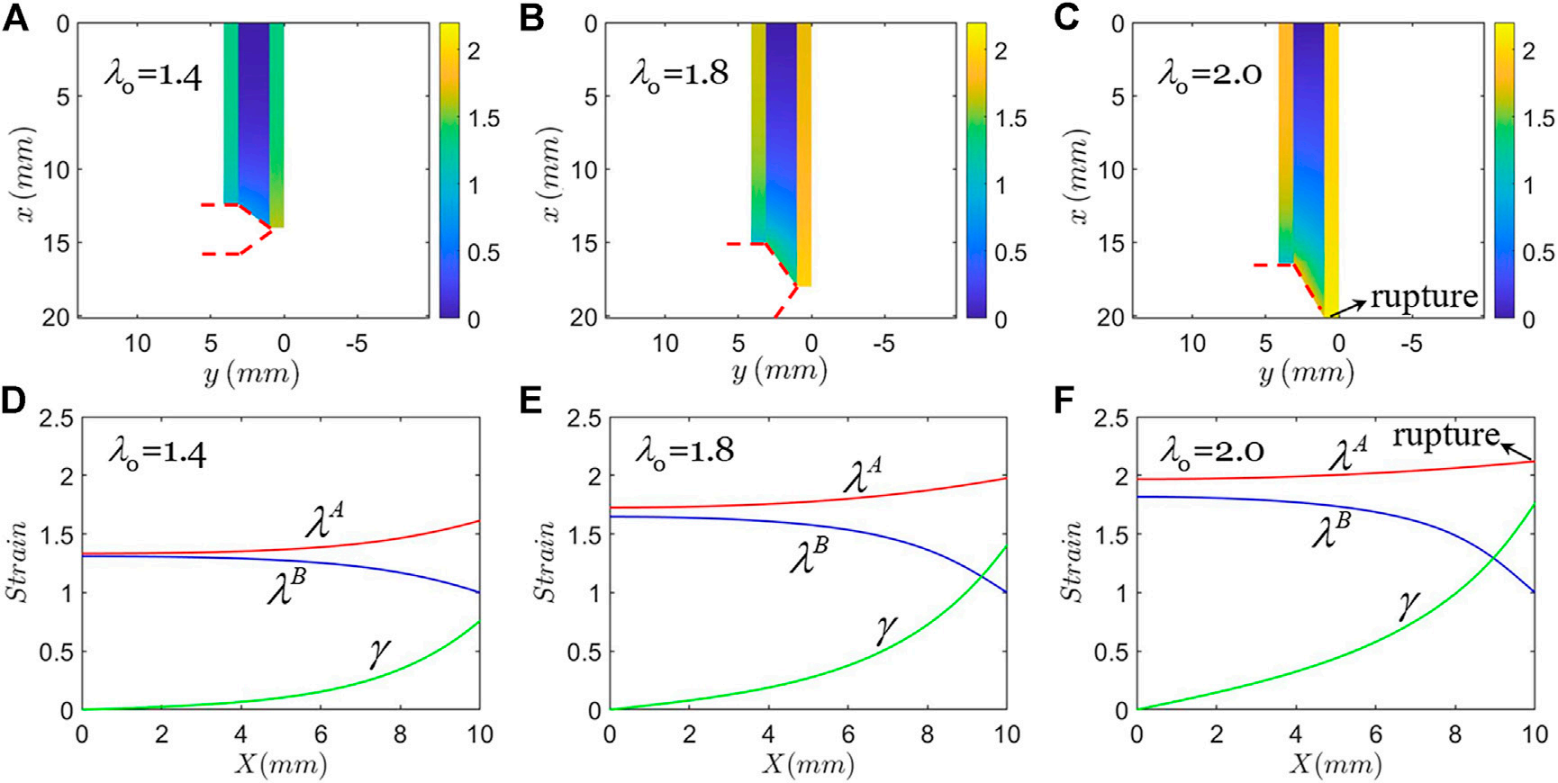
Type A Failure: rupture of hard phase. The applied remote stretch gradually increases until the hard phase breaks, while the interface remains intact. **(A)–(C)** Strain contours plotted in the coordinate of the current state, and **(D)–(F)** strain distribution plotted in the coordinate of the reference state.

Figure 3 shows one type of failure mode: rupture of hard phase. In plotting Figure 3, the material of hard phase is taken to be PDMS of weight ratio m:n=10:1 with modulus of 0.65 MPa, J_lim_=3.5, and rupture stretch $\lambda_{c}=2.12$, and the material of soft phase is taken to be PDMS of weight ratio m:n=15:1 with modulus of 0.17 MPa, and J_lim_=21.8. We set the shear strain of rupture of the soft phase is $\gamma_{c}=2$. When the applied stretch is $\lambda_{0}=1.4$ the crack opens (Figure 3A). When the applied stretch is $\lambda_{0}=1.8$ the crack opens more (Figure 3B). When the applied stretch is $\lambda_{0}=2.017$, the maximum stretch in the hard phase A reaches the rupture stretch, $\lambda_{c}$ (Figure 3C). In this state, the maximum shear strain in the soft phase is $\gamma_{\max}=1.746$, smaller than $\gamma_{c}$. The hard phase A ruptures and the crack can propagate forward. Figures 3D,E,F show the strain distribution in the coordinate of the reference state corresponding to Figures 3A,B,C.

We define the stress concentration factor *K* at rupture as the maximum stress in hard phase A divided by the applied stress in hard phases remote from the crack tip. With the parameters above, *K* is calculated to be 1.45 when the hard phase A is about to rupture. The periodic arrangement of soft phase and hard phase significantly reduces the stress concentration at the crack tip. Compared to the case of linear elasticity, the stress concentration factor is on the same order (Hui et al., 2019). For the stretchable composite analyzed herein, we find that *K* varies slowly as the height of the composite *H* changes or as the applied total stretch $\lambda_{0}$ changes. For the non-stretchable composite analyzed using linear elasticity theory of small deformation (Hedgepeth, 1961), K is intendent of *H* and $\lambda_{\text{0}}$.

**FIGURE 4:**
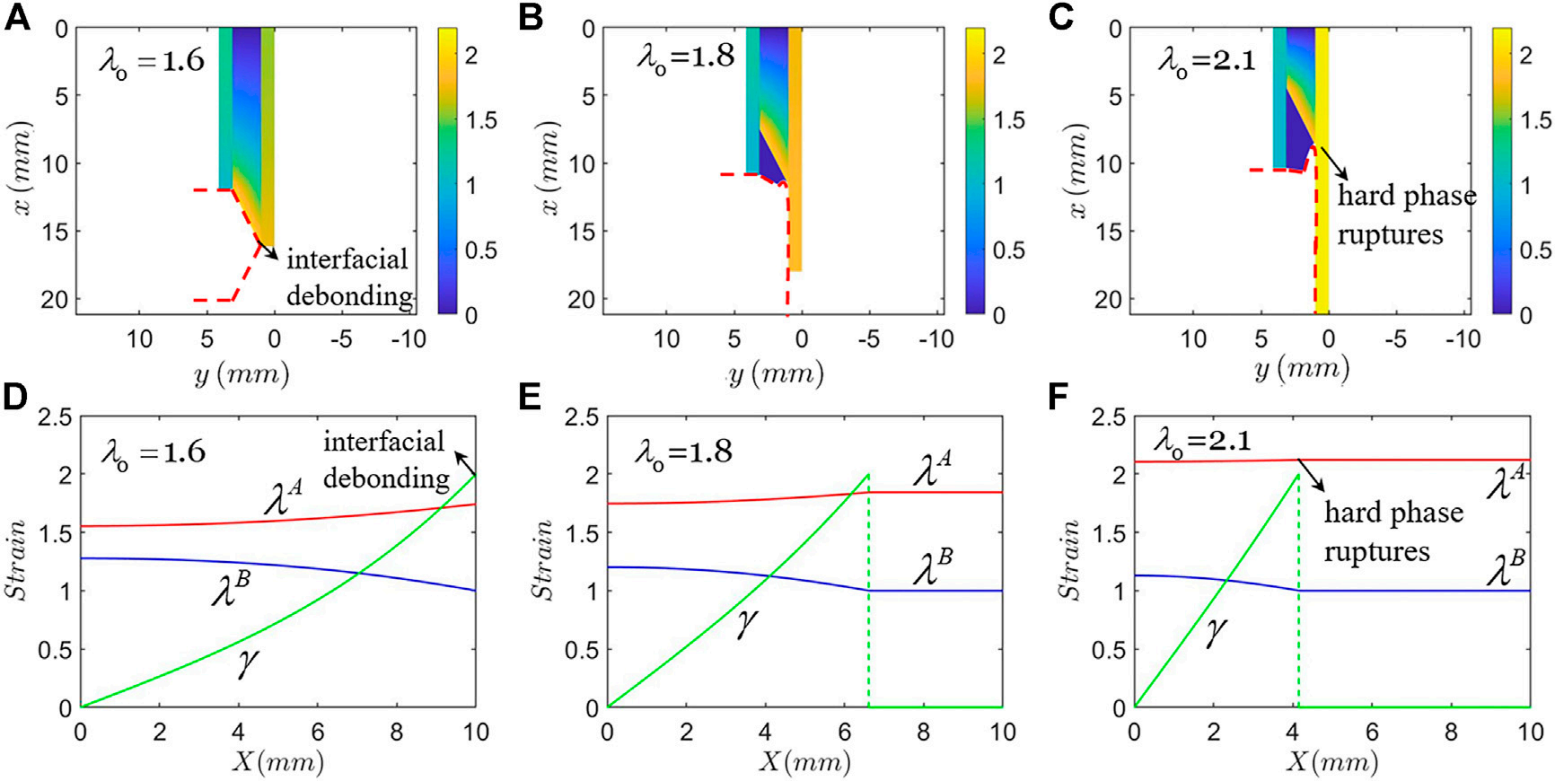
Type B Failure: interfacial debonding. The applied remote stretch gradually increases until the interface debonds and the crack kinks, while the hard phase remains unbroken. Further stretch eventually breaks the hard phase. **(A)–(C)** Strain contours plotted in the coordinate of the current state. **(D)–(F)** The corresponding strain distribution plotted in the coordinate of the reference state.

Figure 4 shows another type of failure mode: interfacial debonding. In plotting Figure 4, the material of hard phase is the same as that in Figure 3, and the material of soft phase is taken to be the PDMS of weight ratio m:n=30:1 with modulus of 0.034 MPa, and J_lim_=62.2. When the applied stretch is $\lambda_{0}=1.616$, the maximum shear strain in the soft phase reaches $\gamma_{c}=2$ (Figure 4A). The interface between the soft phase and the hard phase A debonds and the crack kinks. In this state, the maximum stretch in the hard phase A is $\lambda_{\max}^{A}=1.742$, smaller than $\lambda_{c}$. When the applied total stretch further increases to $\lambda_{0}=1.8$, the shear strain of more material particles reaches $\gamma_{c}=2$, so that the crack propagates along the interface (Figure 4B). We set the shear strain of the debonding zone to be zero, as shown in the dark blue regions. The boundary of the debonding zone is determined by the debonding criterion $\gamma_{c}=2$. The shape of the debonding zone is undetermined in the present analysis and does not affect the stress field of the composite. When the applied total stretch increases to $\lambda_{0}=2.116$, the maximum stretch in the hard phase A reaches the rupture stretch, $\lambda_{c}$ (Figure 4C), and the hard phase A ruptures. In this state, the stress concentration factor is *K*=1.02. In this failure mode, the occurrence of interfacial debonding further reduces the stress concentration at the crack tip. The hard phase A finally ruptures as if there was no crack present. Figures 4D,E,F show the strain distribution in the coordinate of the reference state corresponding to Figures 4A,B,C.

## Toughness and Comparison With Experiment

Referring to Figure 1, for the pure shear configuration, that is, the cracked sample with the top and bottom ends clamped, the energy release rate of a pure material *G* is given by G=HW (Rivlin and Thomas, 1953), where *H* is the height of the material, and *W* is the energy density of the material without crack. At the critical condition, the crack propagates and the critical energy release rate defines the fracture toughness, $\Gamma =G_{c}=HW_{c}$. Here, we define the average toughness Γ of the composite by replacing the energy density with a rule of mixture $W=\varphi_{h}W_{h}+\left(1-\varphi_{h}\right)W_{s}$,

$$\Gamma =H\left[-\varphi_{h}\frac{\mu_{h}J_{\lim h}}{2}\ln\left(1-\frac{{\left(\lambda_{0}^{cr}\right)}^{2}+\frac{2}{\lambda_{0}^{cr}}-3}{J_{\lim h}}\right)-\left(1-\varphi_{h}\right)\frac{\mu_{s}J_{\lim s}}{2}\ln\left(1-\frac{{\left(\lambda_{0}^{cr}\right)}^{2}+\frac{2}{\lambda_{0}^{cr}}-3}{J_{\lim s}}\right)\right],$$ (8)

where $\varphi_{h}$ is the volume fraction of hard phase, $\lambda_{0}^{cr}$ is the critical applied stretch when hard phase A ruptures for both failure modes.

**FIGURE 5:**
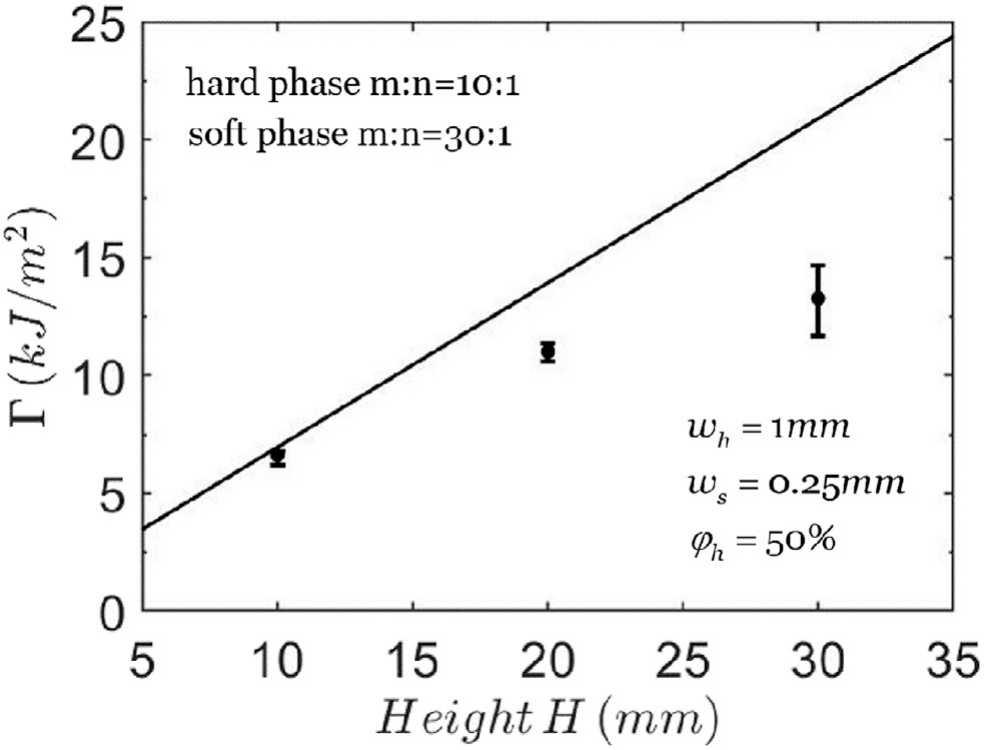
The toughness of composites with different heights. The solid line represents the theoretical prediction and the points with error bars represent the experimental data (Wang et al., 2019).

Figure 5 shows that the calculated average toughness Γ of the composite increases almost linearly with the height of the composite, *H*. The understanding is the following. The existence of soft phase greatly de-concentrates stress at the crack tip so that the critical total stretch $\lambda_{0}^{cr}$ is close to the rupture stretch of the hard phase $\lambda_{c}$. Therefore, the average energy density of the composite *W* approaches a constant irrelevant of the presence of crack and Γ becomes linear with *H*. This feature is in sharp contrast to pure elastic materials, such as the hard phase or the soft phase alone, for which the toughness is independent of the height of sample. Also included are the points with error bar from the experimental data in literature (Wang et al., 2019). The toughness in experiments is obtained by measuring the average energy density *W* using a composite without crack and measuring the critical applied stretch $\lambda_{0}^{cr}$ using a composite with a crack. In plotting the curves of theoretical predictions, the geometric parameters are the same as those used in experiment and the material parameters are from Table 1, because the material parameters reported in the experiment are not complete to use.

**FIGURE 6:**
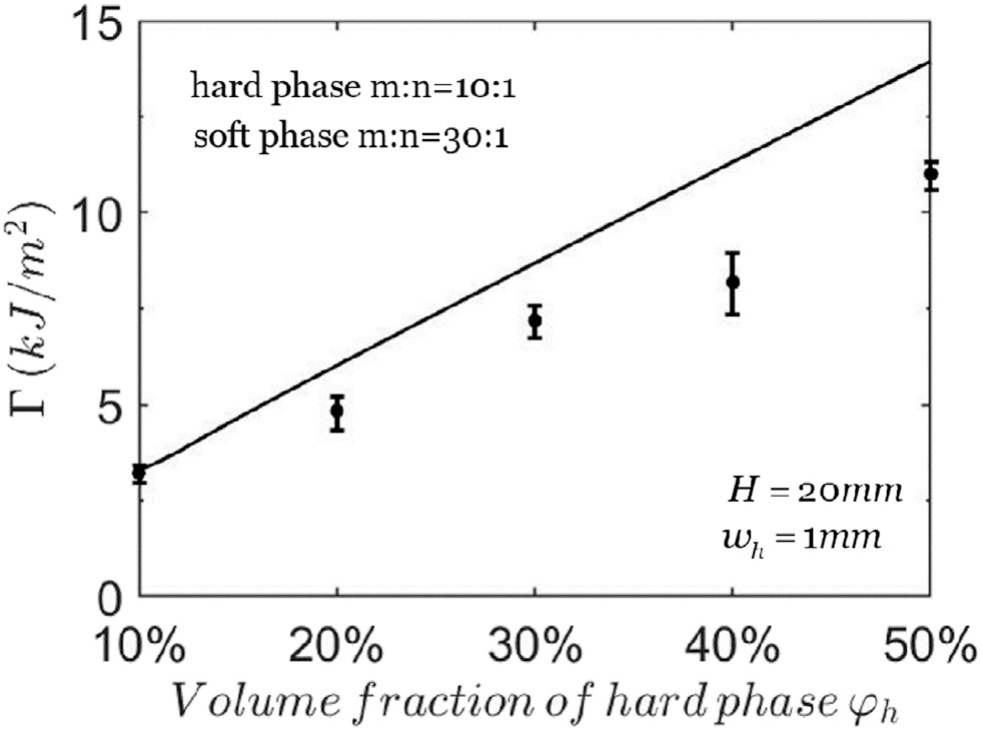
The toughness of composites with different volume fractions of hard phase. The solid line represents the theoretical predictions and the points with error bars represent the experimental data (Wang et al., 2019).

Figure 6 shows that the calculated average toughness Γ of the composite increases almost linearly with the volume fraction of hard phase $\varphi_{h}$. The width of hard phase is fixed at $w_{h}=1\;\mathrm{mm}$. The understanding is the following. When the volume fraction $\varphi_{h}$ changes from 10 to 50%, both the stress concentration factor *K* and the critical total stretch $\lambda_{0}^{cr}$ change slightly. The energy density in hard phase is larger than that in soft phase. Therefore, according to Eq. 8, the average toughness is linear with the volume fraction $\varphi_{h}$ if $\lambda_{0}^{cr}$ remains unchanged.

**FIGURE 7:**
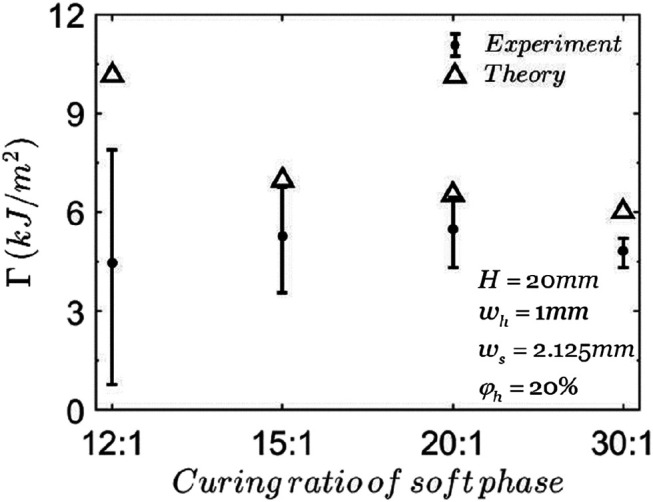
The fracture toughness of composites with different curing ratios of soft phase. The triangles represent the theoretical predictions and the points with error bars represent the experimental data (Wang et al., 2019).

Figure 7 shows the effect of the modulus of soft phase. When the modulus of soft phase is 0.034 MPa (curing ratio m:n=30:1), the stress concentration at the crack tip is small, *K*=1.02. When the modulus of soft phase is 0.40 MPa (curing ratio m:n=12:1), the stress concentration at the crack tip becomes larger, *K*=1.61, which decreases the critical total stretch, $\lambda_{0}^{cr}$ and tends to decrease the average toughness, Γ. On the other hand, when the modulus of soft phase increases, the elastic energy stored in the composite at the same stretch ratio increases, which tends to increase the average toughness. These two effects influence the toughness simultaneously.

According to our calculations, when the soft phase is softer, it is more likely to have Type B failure: interfacial debonding. The experiments show that when the curing ratios of the soft phase are 20:1 and 30:1, the interfacial debonding is observed first in the composite and finally the hard phase ruptures. When the curing ratios of the soft phase are 15:1 and 12:1, only rupture of hard phase is observed in the composite without interfacial debonding.

The theoretical analysis in this work has several idealizations. First, the interfacial bonding strength between hard phase and soft phase of different curing ratios should be different and affects the failure mode of the composite. The criterion of interfacial debonding may depend on the modulus of the two adherends, the method of adhesion, the thickness of the adhesion layer and the local stress concentration. To avoid these complications, throughout the paper we use a simple criterion to describe the interfacial debonding, $\gamma_{c}=2$. Second, only three layers close to the crack tip are used to analyze the deformation fields of the composite and the effects from other layers are neglected. As the modulus of soft phase and hard phase become closer, the effects from the nearby layers become more prominent. Third, the stress state in the composite strongly depends on the geometric parameters. Throughout the paper, we assume the soft phase under simple shear, and the whole composite under plane stress state. To carefully examine all the effects above, a more refined finite element model is needed.

## Conclusion

We establish a theoretical model to analyze the large deformation and failure of the stretchable composite with periodically arranged hard phase and soft phase. We find that the stress concentration at the crack tip is much reduced in such composite. We analyze two types of failure modes: rupture of hard phase and interfacial debonding. Both failure modes can greatly improve the fracture resistance by stress de-concentration. We calculate the average toughness of the composite with different physical and geometric parameters. We find that the toughness of the composite increases almost linearly with the height of the composite and the volume fraction of hard phase. If the interfacial bonding between the hard phase and the soft phase is too strong, the stress concentration is amplified. A relatively weak interface would be helpful to increase the fracture toughness. Increasing the modulus of the soft phase increases the elastic energy storage in the soft phase but also increases the stress concentration, these two coupling effects influence the toughness simultaneously. The experimental results in literature agree with our theoretical predictions very well. This paper illustrates the toughening mechanism of the unidirectional stretchable composite and may provide a tool to optimize similar stretchable composites in practical applications.

## Data Availability

The original contributions presented in the study are included in the article/Supplementary Material, further inquiries can be directed to the corresponding author.
