# Supplementary material for: Toughening Mechanism of Unidirectional Stretchable Composite
Source: Front Robot AI. 2021 Apr 30;8:673307. doi: 10.3389/frobt.2021.673307 (PMC8120101; doi:10.3389/frobt.2021.673307)
Supplement: Supplementary file 1 [file Image1.pdf]

Supplementary Material

Supplementary Figure

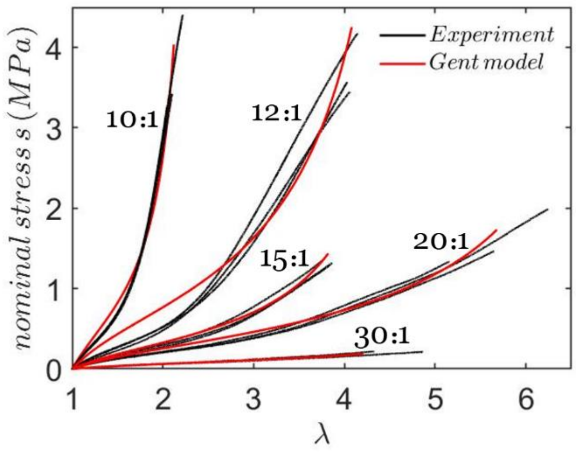

Figure S1. Stress-stretch curves of PDMS samples with different curing ratios (curing ratio m:n =10:1, 12:1, 15:1, 20:1, 30:1) under uniaxial tension and the fitting curves using the Gent model.
